# Characterization of the Interaction of Neuropathy Target Esterase with the Endoplasmic Reticulum and Lipid Droplets

**DOI:** 10.3390/biom9120848

**Published:** 2019-12-09

**Authors:** Pingan Chang, Ling He, Yu Wang, Christoph Heier, Yijun Wu, Feifei Huang

**Affiliations:** 1 Chongqing Key Laboratory of Big Data for Bio-Intelligence, School of Bio-information, Chongqing University of Posts and Telecommunications, Chongqing 400065, China; S190502002@stu.cqupt.edu.cn (L.H.); yuwangbio@gmail.com (Y.W.); 2 Institute of Molecular Biosciences, University of Graz, 8010 Graz, Austria; christoph.heier@uni-graz.at; 3 Laboratory of Molecular Toxicology, State Key Laboratory of Integrated Management of Pest Insects and Rodents, Institute of Zoology, Chinese Academy of Sciences, Beijing 100101, China; wuyj@ioz.ac.cn

**Keywords:** neuropathy target esterase, PNPLA6, lipid droplet, endoplasmic reticulum, triacylglycerol, lysophospholipase

## Abstract

Neuropathy target esterase (NTE) is an endoplasmic reticulum (ER)-localized phospholipase that deacylates phosphatidylcholine (PC) and lysophosphatidylcholine (LPC). Loss-of-function mutations in the human *NTE* gene have been associated with a spectrum of neurodegenerative disorders such as hereditary spastic paraplegia, ataxia and chorioretinal dystrophy. Despite this, little is known about structure–function relationships between NTE protein domains, enzymatic activity and the interaction with cellular organelles. In the current study we show that the C-terminal region of NTE forms a catalytically active domain that exhibits high affinity for lipid droplets (LDs), cellular storage organelles for triacylglycerol (TAG), which have been recently implicated in the progression of neurodegenerative diseases. Ectopic expression of the C domain in cultured cells decreases cellular PC, elevates TAG and induces LD clustering. LD interactions of NTE are inhibited by default by a non-enzymatic regulatory (R) region with three putative nucleotide monophosphate binding sites. Together with a N-terminal TMD the R region promotes proper distribution of the catalytic C-terminal region to the ER network. Taken together, our data indicate that NTE may exhibit dynamic interactions with the ER and LDs depending on the interplay of its functional regions. Mutations that disrupt this interplay may contribute to NTE-associated disorders by affecting NTE positioning.

## 1. Introduction

Neuropathy target esterase (NTE)—also termed patatin-like phospholipase domain containing (PNPLA) 6—was identified as a principal target of organophosphates that cause a delayed neuropathy characterized by the degeneration of long axons and the paralysis of the lower limbs [1,2,3,4]. Functional studies revealed that NTE acts as (lyso)phospholipase with a preference for phosphatidylcholine (PC) and lysophosphatidylcholine (LPC) and that organophosphate-induced neuropathy was most likely caused by the inhibition of NTE activity and a resultant disruption of membrane homeostasis [5,6,7,8,9].

NTE is mainly expressed in the nervous system but also in several non-neuronal tissues such as kidney, liver and testis [10]. The protein plays an essential role in mammalian embryo development [10,11]. Global disruption of the *Nte*/*Pnpla6* gene in mice causes embryonic death due to placental failure and impaired vasculogenesis [11]. Apart from this, NTE is also required for neuronal development, including adult axon maintenance and glial ensheathment of Remak fibers [12,13]. Brain-specific deletion of NTE induces neurodegeneration [14], which is likely caused by defective membrane and ER homeostasis [15]. The importance of NTE for neurobiology is emphasized by the observation that loss-of-function mutations in the human *NTE*/*PNPLA6* gene are linked to several complex neurodegenerative syndromes, such as Motor neuron disease (MND), Hereditary Spastic Paraplegia 39 (SPG39), Boucher-Neuhäuser syndrome, Gordon-Holmes syndrome, cerebellar ataxia, Oliver-McFarlane syndrome and Laurence-Moon syndrome [16,17,18,19,20,21].

**Figure 1:**
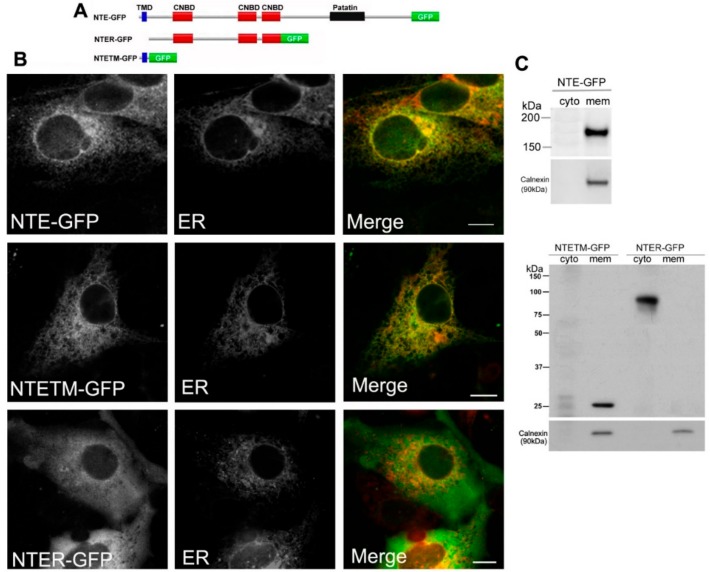
Functional contribution of the N-terminal transmembrane (TM) domain and regulatory (R)-region to ER targeting of neuropathy target esterase (NTE). (**A**) Domain architecture of NTE and the variants used in this experiment. (**B**) Subcellular distribution of NTE-GFP, NTETM-GFP and NTER-GFP in COS-7 cells. COS-7 cells were co-transfected with NTE-GFP, NTETM-GFP, NTER-GFP and the ER marker ER-DsRed as indicated on each panel for 48 h and then visualized live by confocal microscopy. Scale bar, 10 μm. Figures are representative of three separate experiments. (**C**) Subcellular distribution of NTE-GFP, NTETM-GFP and NTER-GFP in transfected mammalian cells. After transfection for 48 h, cells were harvested, homogenized and fractionated into membrane (mem) and cytosolic (cyto) fractions. The cytosolic and membrane fractions were further subjected to Western blotting analysis with an anti-GFP antibody. Migration of molecular mass standard proteins is indicated left of the figure.

Human NTE is a polypeptide of 1327 amino acids and has two functional regions: The amino-terminal region (amino acids 1-680) contains a N-terminal transmembrane domain (TMD) and a regulatory (R) domain with three putative cyclic nucleotide-binding domains (CNBDs); the carboxyl-terminal catalytic (C) region (amino acid 681-1327) is characterized by the presence of a patatin domain predicted to mediate enzyme activity (Figure 1A) [5]. Initial studies revealed that the catalytic activity indeed resides mainly in the C-terminal region of the polypeptide. Mutagenesis studies assigned critical catalytic roles to residues Ser966, Asp960 and Asp1086, which were proposed to constitute a catalytic triad [5,9]. A NTE fragment encompassing these residues termed NTE est erase region (NEST; amino acids 727-1216) potently hydrolyzed several membrane lipids in vitro [6] suggesting that the C domain acquires catalytic competence also in the absence of N-terminal regions. 

Although N-terminal TM and R domains are largely dispensable for catalytic competence they may play critical roles in subcellular localization of NTE. The N-terminal TMD was proposed to anchor NTE in the ER membrane exposing the majority of the polypeptide including the C domain to the cytosol [5,14]. In addition, the C domain was proposed to promote ER association via hydrophobic areas of the patatin domain [22]. The contribution of the R-region to the subcellular positioning of NTE is less clear [5,22]. There are three putative CNBDs in the R-region but no evidence has been provided that cyclic AMP directly binds NTE [23]. The R region of swiss cheese (sws), the *Drosophila* orthologue of mammalian NTE, interacts with the C3 catalytic subunit of cAMP activated protein kinase (PKA-C3) and affects PKA activity [24]. This suggests that the R region confers non-enzymatic functions of NTE. However, how the R region regulates subcellular positioning and activity of NTE remains largely elusive. 

Recently, a number of HSP- and MND-related proteins, such as seipin, spartin and spastin, have been linked to the biology of lipid droplets (LDs). LDs are cellular storage organelles for neutral lipids like triacylglycerol (TAG) and contribute to membrane and energy homeostasis and cellular stress responses [25,26,27]. *De novo* formation of LDs occurs at the ER [26]. Since NTE is an ER-localized phospholipase and its dysfunction is linked to HSP we asked if NTE interacts with LDs and if NTE affects LD biology. In the current study, we further characterized the contribution of protein domains to the interaction of NTE with ER and LDs and its catalytic activity. 

## 2. Results

### 2.1. The N-Terminal TMD but Not the R-Region of NTE Localizes to the ER

Previous studies showed that the N-terminal TMD facilitates association of NTE with ER membranes [5]. If the R-region contributes to ER targeting of NTE is less clear. We first analyzed the subcellular distribution of full length NTE, the N-terminal TMD and the R-region fused to GFP (NTE-GFP, NTETM-GFP and NTER-GFP, Figure 1A). As shown in Figure 1B, NTE-GFP and NTETM-GFP displayed a reticular staining pattern, which was completely colocalized with an ER marker. In contrast, NTER-GFP exhibited a diffuse cytosolic distribution and did not coincide with the ER. These results show that the N-terminal TMD but not the R-region is sufficient to target cytosolic GFP to the ER membrane. To further confirm these results, we performed subcellular fractionation experiments. Western blotting analysis of cytosolic and membrane fractions prepared from COS-7 cells expressing NTE-GFP, NTETM-GFP and NTER-GFP revealed that NTE-GFP and NTETM-GFP were exclusively present in the membrane fraction whereas NTER-GFP was recovered predominantly from the cytosolic fraction (Figure 1C). The presence of calnexin, an integral ER-membrane protein, was detected to assess the purity of the fractions. As shown in Figure 1C, calnexin was only present in the membrane fraction. Thus, the N-terminal TMD of NTE localized to the ER membrane and was sufficient for ER targeting of NTE, while the R-region did not exhibit ER localization.

### 2.2. The C Domain of NTE Binds to LDs

**Figure 2:**
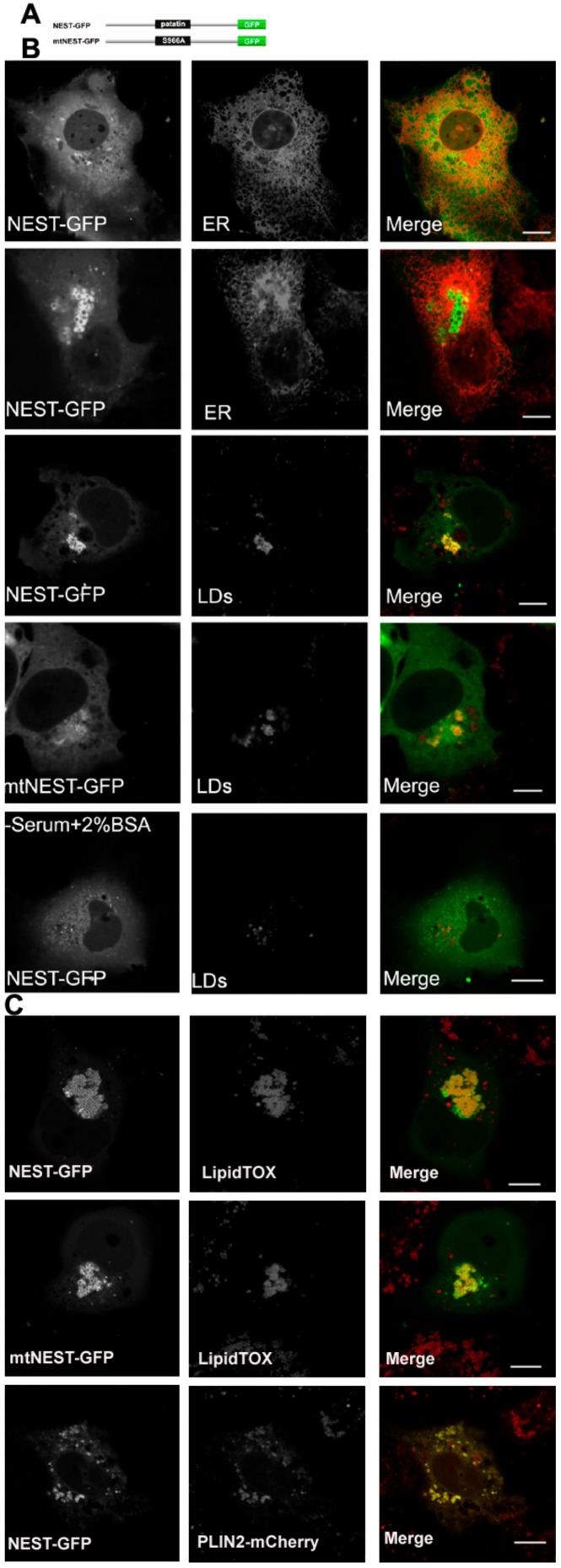
NTE esterase domain (NEST) localizes to LDs. (**A**) Domain architecture of a NEST and a S966A NEST mutant (mtNEST)construct used in this experiment. (**B**) NEST-GFP partially localizes to LDs in COS-7 cells without OA-loading. COS-7 were co-transfected with NEST-GFP and the ER marker, ER-DsRed or transfected alone with NEST-GFP or mtNEST-GFP as indicated. Within 48 h, living cells were imaged by confocal microscopy. To detect the distribution of NEST-GFP, mtNEST-GFP and LDs, COS-7 cells were fixed and incubated with LipidTOX™ Deep Red to stain LDs and then visualized by confocal fluorescence microscopy. (**C**) Increased fatty acid flux stimulates NEST binding to LDs. COS-7cells expressing NEST-GFP or mtNEST-GFP were treated with OA overnight. LDs were labeled by LipidTOX™ Deep Red or by co-expression of PLIN2-mCherry. Colocalization of NEST-GFP, mtNEST-GFP and LDs was visualized by confocal microscopy. Scale bar = 10 μm. Figures are representative of three separate experiments.

The C domain of NTE (681-1327 residues), also termed NEST, has previously been shown to distribute between ER and cytosol [5]. It has been suggested that the C domain contributes to ER association of NTE [5]. We re-evaluated the subcellular localization of NEST-GFP and found that it exhibits a diffuse cytosolic distribution in most cells but does not co-localize with the ER (Figure 2B). In a few cells NEST-GFP labeled vesicles, which also did not coincide with the ER (Figure 2B). A cellular staining for neutral lipids revealed that the NEST-positive vesicles represent LDs. Whether the association of NEST with LDs was related to its catalytic activity was further addressed. As shown in Figure 2B, the inactive S966A NEST-GFP mutant (mtNEST-GFP) still localized to LDs, indicating that the localization of NEST to LDs reflects a non-enzymatic property. When cells were depleted of LDs by culturing them in serum free medium with BSA, NEST-GFP was shifted towards the cytoplasm. Conversely, when LD formation was stimulated by exogenous oleic acid (OA) both NEST and the catalytically inactive mtNEST accumulated at LDs (Figure 2C). Moreover, LD clustering was observed in NEST-GFP and mtNEST-GFP-expressing cells. To further confirm association of NEST-GFP with the LD surface, LDs were labeled by the expression of the *bona fide* LD protein PLIN2 fused to mCherry. As shown in Figure 2C, NEST-GFP strongly colocalized with PLIN2-mCherry, indicating that NEST-GFP localized to the LD surface. Taken together, these results suggest that the C terminal domain of NTE has an intrinsic affinity for the LD surface and induces LD clustering independent of its enzymatic activity.

### 2.3. NTE Does Not Localize to LDs

**Figure 3:**
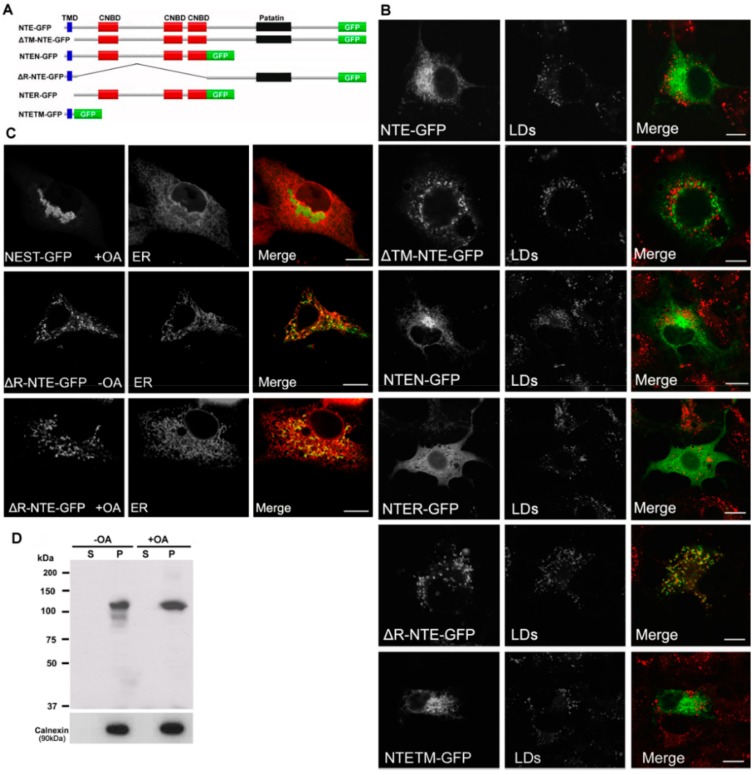
Functional contribution of NTE protein domains to LD targeting. (**A**) Domain architecture of NTE and its variants used in this experiment. (**B**) Subcellular distribution of NTE variants and LDs. COS-7 cells were transfected with NTE-GFP or truncated NTE-GFP mutants, incubated with FAs to induce LD formation and analyzed by confocal fluorescence microscopy. LDs were visualized using the neutral lipid stain HSC LipidTOX™ Deep Red with PLIN2-mCherry. Bar size: 10 µm. (**C**) Subcellular distribution of NEST-GFP, ∆R-NTE-GFP and ER. NEST-GFP or ∆R-NTE-GFP was expressed in COS-7 cells loaded with OA or not as indicated. The ER was marked by co-expression of ER-DsRed. Images were acquired by confocal fluorescence microscopy. Scale bar, 10 µm. (**D**) Subcellular distribution of ∆R-NTE-GFP in COS-7 cells in the absence or presence of OA. After transfection for 24 h, cells were incubated in the absence or presence of OA for 24 h before being subjected to subcellular fractionation. The soluble (S) and particulate (P) fractions were subjected to immunoblotting analysis using antibodies against GFP and Calnexin.

We next investigated whether full length NTE also interacts with LDs. In contrast to NEST-GFP, NTE-GFP showed a reticular staining pattern and did not decorate LDs when expressed in COS-7 cells (Figure 3B). Similar results were obtained using human neuroblastoma SH-SY5Y cells (Supplementary Figure S1). To exclude a potential effect of GFP on the distribution of NTE, we observed the localization of endogenous NTE and LDs in SH-SY5Y cells. NTE still displayed a reticular staining pattern and did not localize to LDs even after increasing LD formation by exogenous OA supplementation (figure not shown). Whether the subcellular localization of NTE was related to its catalytic activity was investigated by means of the inactive S966A NTE mutant (mtNTE). However, as shown in Supplementary Figure S2, also mtNTE-GFP localized in a reticular ER-like pattern and did not accumulate at LDs.

We further explored the relationship between the protein domains of NTE and LD localization using truncated NTE mutants (Figure 3A). Similar to NTE, NTEN-GFP containing the N-terminal TMD and R-region still displayed a typical ER staining after OA loading and did not localize to LDs (Figure 3B). Likewise, NTETM-GFP and NTER-GFP, which represent the isolated TM and R domains, did not decorate LDs (Figure 3B) suggesting that N-terminal domains do not contain LD targeting information. Interestingly, deletion of the R-region (ΔR-NTE-GFP) was sufficient to provoke LD interaction of the NTE polypeptide. ΔR-NTE-GFP concentrated close to LDs in the cup-like or semi-ring shape, which was clearly different from the ring-shaped structures labeled by NEST-GFP (Figure 3B). Conversely, LD targeting of the C domain was fully prevented by the presence of the R domain (ΔTM-NTE-GFP) even in the absence of the critical N-terminal TMD. This suggests that the presence of the R domain restricts access of the C domain to the LD. 

The LDs in ΔR-NTE-GFP-expressing cells were relatively separated, which was distinct from the clustered LDs in NEST-GFP-expressing cells (Figure 3B), suggesting that the N-terminal TMD may block the clustering of LDs by targeting the protein to the ER. To address this, we further assessed the colocalization of ΔR-NTE-GFP and NEST-GFP with the ER. NEST-GFP associated with the clustering LDs and did not localize to the ER after OA loading (Figure 3C). In contrast, ΔR-NTE-GFP was localized to the ER but aggregated at specific ER domains (Figure 3C). After OA loading, the signal of ΔR-NTE-GFP still coincided with that of the ER marker but appeared aggregated in a dot- or semiring-like shape suggesting that in the absence of the R domain the NTE polypeptide concentrates in ER domains close to LDs (Figure 3C). The localization of ΔR-NTE-GFP to the ER was further confirmed by subcellular fractionation and immunoblotting analysis. ΔR-NTE-GFP was exclusively associated with the membrane/pellet fraction whether cells were incubated with OA or not (Figure 3D). Thus, the presence of the N-terminal TMD firmly localized ΔR-NTE to the ER but permitted LD interactions of the polypeptide.

### 2.4. NEST but Not NTE, is Associated with Pre-Existing LDs after Starvation

**Figure 4:**
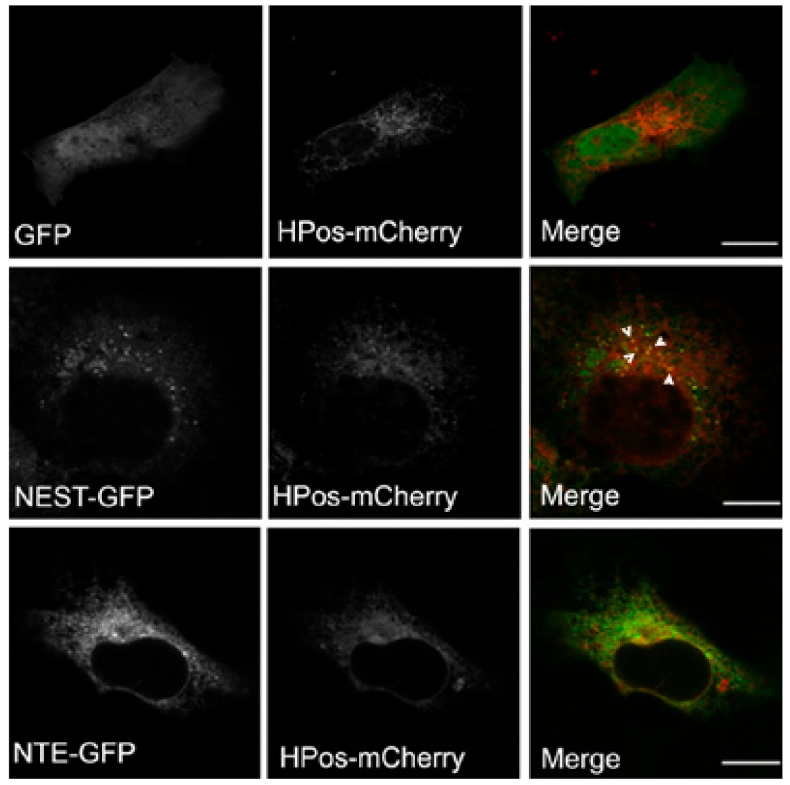
NEST but not NTE co-localizes with the pre-LD marker HPos. COS-7 cells co-expressing GFP, NTE-GFP or NEST-GFP and HPos-mCherry were starved for 24 h. NEST-GFP is present in HPos positive puncta upon starvation as indicated by white arrowheads. NTE does not co-localize with pre-LDs. Scale bar = 10 µm. Figures are representative of three separate experiments.

LDs form in ER microdomains and originate from pre-existing LDs (pre-LDs), which are characterized by a core of neutral lipids that are resistant to starvation [28]. Upon fatty acids loading, neutral lipids are first deposited in pre-LDs to stimulate LD formation. Pre-LDs can be labeled using the model peptide HPos [28]. The relationship of NEST with pre-LDs was investigated to further explore the connection between NEST and LDs. After serum-starvation HPos-mCherry was distributed in ER-like structures associated with small dots indicating pre-LDs. GFP alone displayed a diffuse distribution in the whole cell, which was HPos-negative (Figure 4). NEST-GFP-expressing cells exhibited a large number of small GFP-puncta, some of which were HPos-positive (Figure 4). In contrast, NTE-GFP exhibited a reticular staining pattern, which did not colocalize with pre-LDs labeled by HPos-mCherry (Figure 4). These data indicated that NEST but not NTE associates with pre-LDs defined by HPos labeling after starvation.

### 2.5. Neither the Patatin Domain nor the Putative TM Region in NEST Localize to LDs

**Figure 5:**
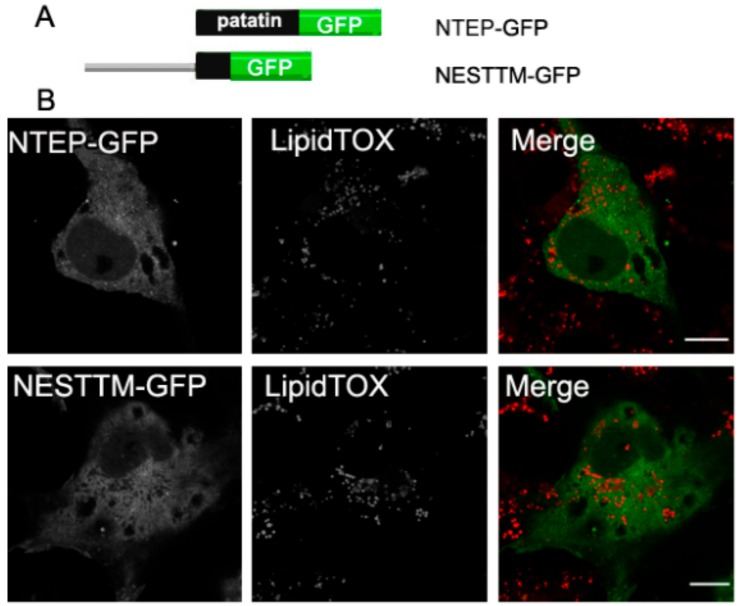
Neither the patatin domain nor putative TM regions in NEST localize to LDs. (**A**) Schematic overview of the patatin domain and putative TM domains in NEST tagged with GFP, NTEPP-GFP and NESTTM-GFP. (**B**) Subcellular localization of NEST truncation variants and LDs. COS-7 cells expressing NTEP-GFP or NESTTM-GFP were loaded with OA for 16 h and then fixed and incubated with LipidTOX Deep Red to stain LDs. Colocalization of proteins and the LDs was visualized by confocal laser scanning microscopy. Scale bar = 10 µm. Figures are representative of three separate experiments.

To further investigate the association of NEST with LDs, we analyzed the polypeptide for potential hydrophobic regions. TMpred analysis indicates three putative TMDs (734–976 residues) in NEST [2,9]. Also, the patatin domain (933–1099 residues) has been suggested to mediate membrane associations due to its hydrophobicity [22]. We therefore expressed these regions separately in COS-7 cells and analyzed the subcellular localization of these constructs. In cells expressing NESTTM-GFP (residues 734–976) or NTEP-GFP (NTE patatin domain) the fluorescence displayed a diffuse cytosolic pattern, which did not concentrate around LDs (Figure 5). Thus, neither the patatin domain nor the putative TM region in NEST is sufficient for LDs targeting.

### 2.6. LD Targeting Does Not Affect the Catalytic Activity of NEST and ΔR-NTE

**Figure 6:**
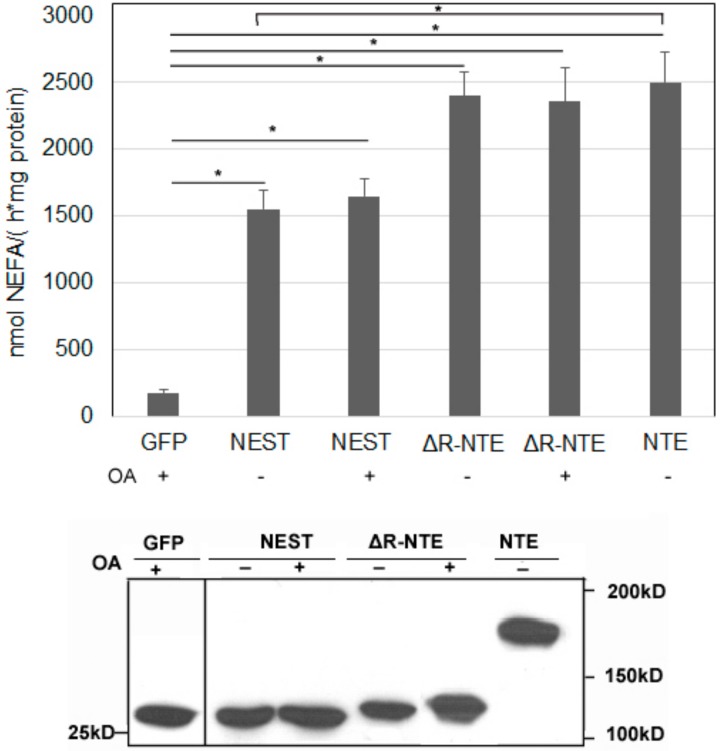
The lysophospholipase activities of neuropathy target esterase (NTE) and its truncated mutants. Lysates of COS-7 cells overexpressing GFP, NEST-GFP, ΔR-NTE-GFP or NTE-GFP in the absence or presence of OA were incubated with LPC as lipid substrate and hydrolytic activities were determined by measuring the released NEFA. GFP served as negative control. Assays were linear with time and protein amount. The Western blot shows comparable protein expression of GFP, NEST-GFP, ΔR-NTE-GFP and NTE-GFP. Normalization was performed by densitometric analyses of the respective expression level detected with an antibody towards GFP. “+” and “-” indicated OA loading or not respectively. Data are presented as means ± SD and are representative of at least three experiments. Asterisk denotes p values: * *p* < 0.05, *n* = 3.

We next tested if the LD localization of NEST and ΔR-NTE alters catalytic activity. LPC is the preferred substrate of NTE [6,7]. As shown in Figure 6, when LPC was used as the substrate, the released non-esterified fatty acids (NEFA) were increased significantly in NEST-GFP, ΔR-NTE-GFP and NTE-GFP expressing cells compared with cells expressing GFP alone. In the absence of exogenous OA, the LPC hydrolysis activity of NEST-GFP was about 60% of NTE-GFP activity, while ΔR-NTE-GFP showed a similar enzymatic activity as NTE-GFP, indicating that deletion of the R-region does not affect the catalytic activity of NTE. Upon OA incubation to promote LD formation the LPC hydrolase activities of NEST-GFP- and ΔR-NTE-GFP-expressing cells were similar when compared with cells cultured without OA. Thus, LD localization of NEST and ΔR-NTE in response to lipid loading does not alter catalytic activity in vitro.

### 2.7. Effect of NTE Activity and Localization on LD Morphology and Triacylglycerol Levels

**Figure 7:**
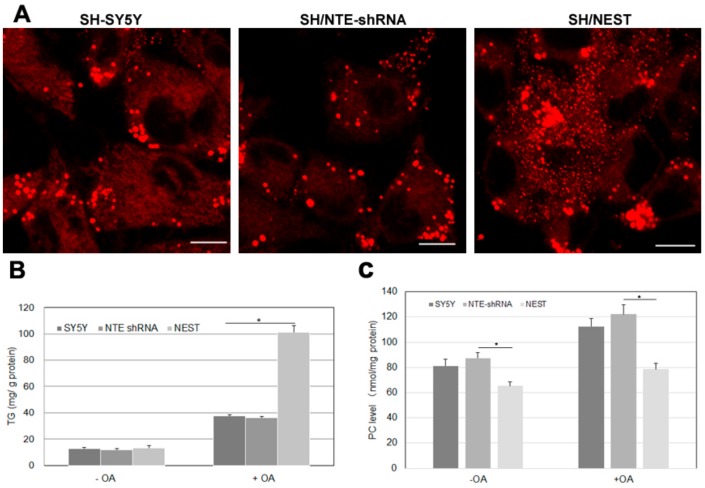
LD morphology and lipid levels upon knockdown of NTE and overexpression of NEST in human neuroblastoma cells. SH-SY5Y control cells, NTE-knockdown cells (SH/NTE-shRNA) and NEST-expressing cells (SH/NEST) were incubated with OA overnight. (**A**) LDs were labeled by LipidTOX Deep Red and visualized by confocal laser scanning microscopy. Scale bar = 10 µm. Figures are representative of three separate experiments. (**B**) and (**C**) Triacylglycerol (TG) and PC levels were measured in control cells (SY5Y), NTE-knockdown cells (NTE shRNA) and NEST-overexpressing cells (NEST) incubated in the absence (−OA) or presence (+OA) of exogenous OA. Data are presented as means ± SD and are representative of at least three experiments. Asterisk indicates p values: * *p* < 0.05, *n* = 3.

NTE is mainly expressed in the nervous system, especially in brain and spinal cord and highly expressed in human neuroblastoma cells [29]. We finally investigated how alterations in NTE expression affect LDs and triacylglycerol levels in human SH-SY5Y cells. We created two stable cell clones: SH/NTE-shRNA cells express shRNA targeting *NTE* and show a ~70% reduction in *NTE* mRNA levels compared to control cells; SH/NEST cells stably express NEST [30]. SH/NTE-shRNA cells exhibited unaltered LD morphology when compared to control cells (Figure 7A). In contrast, expression of NEST-induced a high number of small LDs and LD clusters in the perinuclear region (Figure 7A). In the absence of OA SH/NTE-shRNA and NEST-expressing cells exhibited similar TAG levels as control cells (Figure 7A) The addition of OA increased TAG in all cell lines but to a much higher extent in SH/NEST cells compared to control or SH/NTE-shRNA cells (Figure 7B). PC levels were decreased in SH/NEST cells both, in the absence and presence of exogenous OA but were unaltered in SH/NTE-shRNA cells as compared to control cells (Figure 7C). 

## 3. Discussion

**Figure 8:**
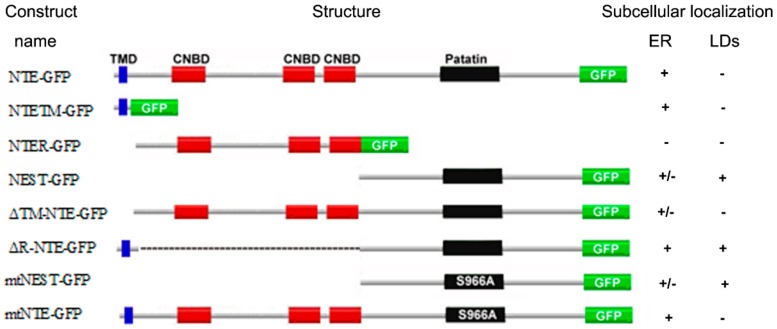
Summary of the subcellular localization of NTE constructs. Localization to the ER or LDs is described as positive (+), whereas failure to localization as negative (−). “+/−” indicates partial localization to the ER. For comprehensiveness, the results of a previous study were included in this summary [5].

NTE has been identified as a patatin domain-containing (lyso)phospholipase with a preference for PC and LPC and pivotal functions in neuronal health [3,4,7,8]. The domain architecture of NTE comprises a N-terminal TMD, a R-region with three putative CNBDs and the C-terminal catalytic region characterized by a patatin domain [5]. In this study we analyzed the contribution of NTE protein domains to the interaction of NTE with the ER and LDs. The localization of the truncation mutants used by us is summarized in Figure 8. For comprehensiveness, we also considered the results of a previous study [5]. Taken together we conclude form these localization patterns that the catalytic C domain of NTE has a high intrinsic affinity for the surface of lipid droplets, which by default is inhibited by the R domain.

In line with previous results our data demonstrate that ER localization of NTE depends on its N-terminal TMD [5]. Deletion of this domain compromises ER localization of NTE. Conversely, the isolated TMD is sufficient to target GFP to the ER. The normal distribution of NTE further depends on the presence of its R domain. Ablation of the R domain renders the polypeptide associated with the ER but promotes its aggregation in specific domains. This aberrant distribution is mediated by the C domain as its ablation restores a normal ER distribution of the polypeptide. Because the C domain is prone to aggregation we speculate that the specific arrangement of TM, R and C domains serves (1) to firmly anchor the protein in the ER and (2) hinder intermolecular associations of the catalytic C domain. 

The deletion of N-terminal ER targeting information uncovered a high intrinsic affinity of the C terminal NEST domain for the LD surface. Even in starved cells a fraction of NEST localized to pre-LDs. Increased LD formation promoted association of NEST with LDs, which was accompanied by a clustering of LDs in the perinuclear region. LD targeting and the induction of LD clusters were independent of the catalytic activity of NEST suggesting that this phenotype was a consequence of intermolecular associations of NEST rather than a change in the LD monolayer induced by the phospholipase activity of the protein. How does NEST associate with the LD surface? There are several known major pathways of protein targeting to LDs [31]. “Class I” LD proteins are embedded in the ER membrane by hydrophobic hairpin motifs and access LDs via ER-LD membrane bridges. “Class II” LD proteins target LD from the cytosol and associate with the LDs surface through amphipathic helices or other hydrophobic domains. Three putative TMDs (734–976 residues) are predicted by TMpred to be present in NEST [2,9]. Moreover, the hydrophobic surface of the patatin domain could contribute to LD targeting [22]. However, our analysis shows that neither the putative TMDs nor the patatin domain is *per se* sufficient for LD targeting. Different algorithms predict additional TMDs in NEST (Supplementary Table S1). Thus, the LD targeting motif for NEST may depend on several hydrophobic domains. However, as the hydrophobic core of LDs does not allow association of bilayer-spanning TMDs it is likely that the hydrophobic stretches in NEST do not represent true TMDs.

In contrast to the isolated NEST domain full length NTE remained ER-associated and did not localize to LDs even upon increased fatty acid flux. Our mutagenesis experiments revealed that this localization is dependent on the R domain, which inhibits LD association of NEST. As discussed above deletion of the N-terminal TMD is sufficient to compromise ER targeting of NTE but—due to the presence of the R region—not sufficient to allow LD targeting of the polypeptide. Conversely, ablation of the R domain permits LD targeting of NTE even in the presence of the N-terminal TMD. The latter constellation renders the polypeptide firmly associated with the ER but allows its concentration close to the LD surface in dot- or hemiring-shaped patterns. The R-region of NTE is characterized by three putative CNBDs that have been highly conserved during evolution. However, evidence that cyclic AMP or other nucleotides directly bind NTE or affect its activity is currently missing [5,23]. Recently, we have shown that elevated cAMP levels promote interactions of the NTE paralogue NRE/PNPLA7 with LDs [32]. This response required the third CNBD of NRE [32]. Whether cyclic nucleotides affect the LD targeting of NTE needs to be further investigated. The R-region in sws, the *Drosophila* orthologue of NTE, has been shown to act as a non-canonical binding partner of PKA-C3 and plays a role in organophosphate-induced behavioral deficits and neurodegeneration [24,33], suggesting that it confers non-enzymatic functions of the protein. Protein-protein interactions or ligand binding may alter the steric constellation of R and C domains. It is therefore possible that via such inputs the R domain releases the catalytic domain of NTE and permits its interaction with other organelles like LDs. However, further studies are needed to confirm this model. 

NTE functions as a phospholipase to regulate PC and LPC homeostasis [12,21,34]. Recently, several enzymes involved in PC and LPC synthesis were implicated in the regulation of LD structure and triacylglycerol levels, such as CTP:phosphocholine cytidylyltransferase (CCT) [35,36,37], phosphatidylethanolamine N-methyltransferase (PEMT) [38] and LPC acyltransferases 1 and 2 [37,39]. For example, altering CCT activity in a macrophage cell line inversely regulated PC and TAG synthesis rates [40]. A similar relationship was observed in our human neuroblastoma cell model when we overexpressed NEST: A decrease in cellular PC levels was accompanied by an increase in TAG. This is in line with a previous study demonstrating that elevated expression of *Drosophila* sws decreased PC and increased TAG in *Drosophila* heads [34]. Conversely, increased PC and decreased TAG levels were observed in *sws* mutant fly heads [21,34]. Together, these observations suggest that FAs liberated from PC by NTE may be re-routed to TAG synthesis. Alternatively, NTE-induced changes in LD structure may affect TAG storage. A drawback of our analysis is that we did not observe alterations in cellular TAG levels or LD structure upon a reduction in *NTE* expression. However, as our silencing approach reduced *NTE* expression by ~70% residual protein may suffice to allow homeostasis of cellular glycerolipid metabolism. Unaltered levels of PC despite reduced *NTE* expression argue in favor of this hypothesis. 

Numerous mutations in the human *NTE*/*PNPLA6* gene have been associated with a spectrum of complex neurodegenerative syndromes including HSP and MND [16,17,18,19,20,21]. Recent studies unveiled close connections between several proteins linked to MND/HSP and LDs, for example, spastin and DDHD2 [25,26,27]. Our finding that the catalytic domain of NTE associates with LD and alters LD structure and cellular TAG may point to a previously unrecognized role of LDs in NTE-associated disorders. Most disease-associated *NTE* mutations were found in the catalytic region and some of these mutations have been linked to decreased NTE activity and altered LPC levels [18,20,41,42,43]. This suggests impaired enzyme activity as the major pathomechanism of NTE-associated disorders. However, several patients suffering from HSP harbor mutations in the R-region of NTE [16,44]. It has been shown that mutations in the R-region also impact NTE function and LPC metabolism [43]. Given the importance of the R-region for subcellular positioning of NTE we speculate that such mutations may affect LD interactions or compromise the distribution of NTE in the ER. 

In summary, our study reveals novel aspects of the structure–function relationships between NTE protein domains, subcellular positioning and enzyme activity. Our results point to a previously unrecognized interaction of NTE with LDs and suggests that mutations in the R region may affect subcellular positioning of NTE. Future studies are clearly warranted to clarify the possible involvement of NTE in LD biology and the relevance of this interaction for NTE-associated neurodegenerative diseases. 

## 4. Materials and Methods

### 4.1. Materials

African green monkey kidney fibroblast-like COS-7 cells and human neuroblastoma SH-SY5Y cells were purchased from the Cell Center of Chinese Academy of Medical Sciences (Beijing, China). The NTE-GFP, ΔR-NTE-GFP ΔTM-NTE-GFP and mtNTE-GFP constructs were generous gifts from Dr. Paul Glynn [5]. The plasmid encoding adipose differentiation-related protein/perilipin 2 (ADRP, PLIN2) tagged with mCherry was constructed in our lab [32]. Plasmid pEGFP-N3 was purchased from Clontech (Palo Alto, CA, USA). A plasmid encoding for CAV1-mCherry was obtained from Addgene (Cambridge, MA, USA). Transfection reagent Lipofectamine 2000 and HCS LipidTOX™ Deep Red neutral lipid stain were purchased from Life Technologies (Groningen, The Netherlands). Cell culture reagents and OA was from Sigma-Aldrich (St. Louis, MO, USA). LPC (1-hydroxy-2-oleoyl-*sn*-glycero-3-phosphocholine) was purchased from Avanti Polar Lipids, Inc. (Alabaster, AL). E1003 triglyceride assay kit was purchase from Applygen Technologies (Beijing, China). Pfu DNA polymerase, *Xho* I, *Eco*R I and *Age* I were purchased from Takara (Dalian, China). Mouse anti-GFP, anti-calnexin monoclonal antibodies, goat anti-mouse IgG HRP are from Santa Cruz Biotechnology (Santa Cruz, CA, USA). Enhanced chemiluminescence (ECL) reagents are obtained from Pierce Biotechnology (Rockford, IL, USA). NEFA-HR(2) kit was from WAKO Chemicals GmbH (Neuss, Germany).

### 4.2. Plasmids Construction

Enhanced green fluorescent fusion proteins, where the N-terminal TMD (residues 1-40, NTETM-GFP), the R-region (residues 41-680, NTER-GFP), the C-region (residues 681-1327, NEST-GFP) and its activity site S966 mutant (mtNEST-GFP, S966A), the patatin domain (residues 933-1099, NTEP-GFP) or the TM region in NEST (residues734-976, NESTTM-GFP) were joined, in-frame, to the N-terminus of the mammalian expression vector pEGFP-N3, were generated using the corresponding primers shown in Table 1. Except for mtNTEST-GFP using mtNTE-GFP as the template, NTE-GFP was used as the template for other NTE truncated mutants. cDNA sequence for coding HPos (a model peptide combined the hydrophobic domain of ALDI with the last 20 residues of caveolin-1) was amplified by PCR using GFP-HPos as the template and cloned into CAV1-mCherry vector to generate HPos tagged with mCherry at the C-terminus, HPos-mCherry [28]. All plasmids were sequenced to confirm the presence of the desired mutations. 

### 4.3. Cell Culture and Transfection

COS-7 cells and SH-SY5Y were cultured in Dulbecco’s modified Eagle’s medium (DMEM) with 10% fetal bovine serum in a 37 °C incubator with 5% CO_2_. SH/NTE-shRNA and SH/NEST cells that stably expresses shRNA to knockdown NTE and overexpresses NEST, respectively, were maintained in DMEM containing 200 μg/mL G418 [30]. Cell transfection was performed using Lipofectamine 2000 transfection reagent. COS-7 cells were co-transfected with NTE-GFP or its variants and the ER marker, DsRed-ER for 48 h to observe the colocalization of proteins with ER. DsRed-ER consists of an ER targeting sequence of calreticulin fused to the N terminus of DsRed and a C-terminal ER retention sequence (KDEL). COS-7 cells were starved in DMEM in the absence of serum for 24 h beginning directly after transfection of NTE or NEST with HPos-mCherry at the same time to observe the association with pre-existing LDs. To stimulate TAG synthesis and LD formation, cells were incubated with 400 μM OA complexed to fat-free BSA for 16 h after 24-h transfection. In contrast, COS-7 cells were cultured in DMEM containing 2% fat-free BSA and free of serum for 24 h to decrease LDs formation after 24-h transfection. 

### 4.4. Membrane Preparation

After transfection, COS-7 cells were washed two times with PBS, scraped into sample tubes and harvested by centrifugation. Cells were then homogenized on ice with 15 passages through a 25-gauge hypodermic needle. Nuclei and cell debris were removed by centrifugation at 1000× *g* for 5 min at 4 °C. The perinuclear supernatant was further centrifuged at 100,000× *g* for 45 min at 4 °C in an Optima^TM^ TLX ultracentrifuge by using a TLA120 rotor (Beckman). The cytosolic fraction was collected and the membrane pellet was resuspended in PBS. Protein concentrations of the fractions was determined with the BCA Kit according to the manufacturer’s instructions. 

### 4.5. Immunoblot Analysis

The samples were mixed with 5 × SDS loading buffer and boiled for 5 min. All the samples were subjected to SDS/PAGE, transferred to PVDF filters and subjected to immunoblotting analysis as described previously [32]. After detection of GFP, blots were stripped and re-probed for calnexin. Antibodies were used at the following dilutions: mouse anti-GFP, 1:4000; anti-calnexin, 1:1000; anti-mouse IgG, 1:10,000.

### 4.6. Fluorescence Microscopy

After transfection for the indicated time, the cells were either subjected to live cell imaging or washed three times with 1 × PBS and fixed with 4% paraformaldehyde for 30 min. For detection of LDs, fixed cells were incubated with HCS LipidTOX Deep Red (1:500 in PBS) for 30 min. Fluorescent images were acquired by confocal scanning microscopy with a Leica SP5 confocal microscope equipped with a Leica HCX 63 × 1.4 NA oil immersion objective. All the presented experiments were repeated independently at least 3 times.

### 4.7. Lysophospholipase Activity Assay

After 48-h transfection, COS-7 cells were collected by washing with ice-cold PBS two times and harvested by scraping centrifugation. Cells were disrupted on ice in lysis buffer (0.25 M sucrose, 1 mM EDTA and 1 mM dithiothreitol) containing proteinase inhibitors by sonication. Then, homogenates were centrifuged at 1000× *g* and 4 °C for 5 min to remove nuclei and cell debris. Protein concentration of the supernatant was determined with the BCA Kit. The supernatant fraction was used to determine lysophospholipase activity as described previously [32]. In brief, 50 µL cell lysates of COS-7 cells overexpressing NTE or truncated variants (15 µg) or GFP (15 µg) were incubated with 50 µL substrate containing 4 mM LPC, 100 mM Bis-tris propane buffer, pH 7.5, 1 mM EDTA and 4 mM CHAPS for 30 min at 37 °C in a water bath. Each reaction was terminated by heat inactivation at 75 °C for 10 min. The released amount of fatty acids was determined using the HR Series NEFA-HR (2) kit according to the manufacturer’s protocol. 

### 4.8. Measurement of TAG and PC Levels

Cells were incubated overnight in the presence or absence of 400 μM OA. Then, cells were washed with PBS for two times and harvested. Cells were then dissolved in 200–400 μL 1% Triton X-100 by sonication. Whole cell lysates were centrifuged at 10,000× *g* for 5 min at 4 °C. The TAG content of the supernatant was determined using an E1003 triglyceride assay kit. Protein concentration was quantified using a Pierce BCA Protein Assay Kit (Thermo, USA). The amount of PC was quantified by our previous methods [45].

### 4.9. Statistical Analysis

Data were generally expressed as mean ± standard deviation (SD) values. Groups of data were compared by one-way ANOVA and by post hoc analysis using the Student–Keuls method. A difference between means was considered significant at *p* < 0.05.

## Figures and Tables

**Table 1:** Primers used to generate NTE constructs.

Construct Name (Coding Sequence)	Primer Sequence (5′-3′)
NTETM-GFP (1-40)	GCGAATTCGCCATGGAGGCTCCGCTGCAAA
	CAGGATCCGGTTTTTGGCACTCGCAGCC
NTER-GFP (41-680)	GCGAATTCGCCATGCCAGCCCCGGATGGCCCCCG
	CAGGATCCCAAGGTGCCCTCGGGAAGCT
NEST-GFP (681-1327)	GCGAATTCGCCATGGGTCACATCAAACGCCGGTAC
	TTGGATCCGGCATCTGTGGCTGAGCCGGG
NESTTM-GFP (734-976)	GCGAATTCGCCATGCTGGCAACTGTGGCAATCCT
	TTGGATCCCGCGTACAACGCTCCGATGA
NTEP-GFP (933-1099)	GCGAATTCGCCATGCTTGTGCTAGGCGGGGGCGG
	TTGGATCCGCGGGCGATGTCCGCTGGCA
mtNEST-GFP (681-1327, S966A)	GCGAATTCGCCATGGGTCACATCAAACGCCGGTAC
	TTGGATCCGGCATCTGTGGCTGAGCCGGG

## Supplementary Materials

The following are available online at https://www.mdpi.com/2218-273X/9/12/848/s1, Figure S1: NTE esterase domain (NEST), but not NTE localized to LDs in human neuroblastoma cells, Figure S2: NTE inactive mutant mtNTE did not localize to LDs, Table S1: Predicted transmembrane domains for neuropathy target esterase.
