# Supplementary material for: Characterization of the Interaction of Neuropathy Target Esterase with the Endoplasmic Reticulum and Lipid Droplets
Source: Biomolecules. 2019 Dec 9;9(12):848. doi: 10.3390/biom9120848 (PMC6995513; doi:10.3390/biom9120848)
Supplement: Supplementary file 1 [file biomolecules-09-00848-s001.pdf]

Supplementary Materials

Table S1. Predicted transmembrane domains for neuropathy target esterase.

Analysis software	Number of TMD	Location of TMD
TMHMM	1	3-31
TMpred	4	11-32,734-753,925-943,956-976
SOSUI	1	10-32
HMMTOP	1	9-32
TopPred	5	12-32,733-753,922-942,956-976,999-1019

Figure S1.

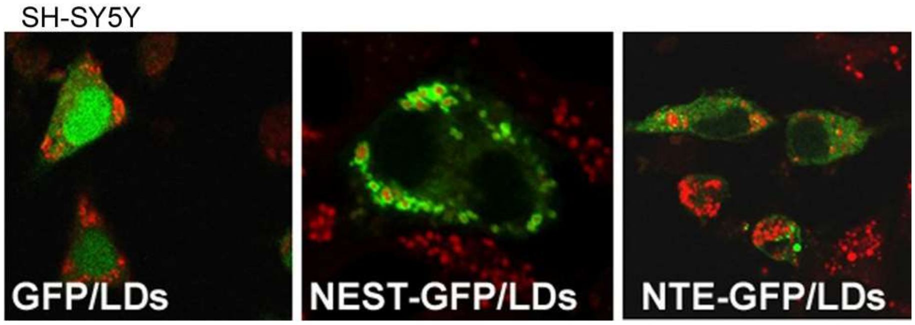

Figure 1. NTE esterase domain (NEST), but not NTE localized to LDs in human neuroblastoma cells. SH-SY5Y cells expressing GFP, NEST-GFP or NTE-GFP were treated with OA overnight. LDs were labeled by LipidTOX Red. Colocalization of GFP-fused proteins with LDs was visualized by confocal laser scanning microscopy. Figures are representative of three separate experiments.

Figure 2.

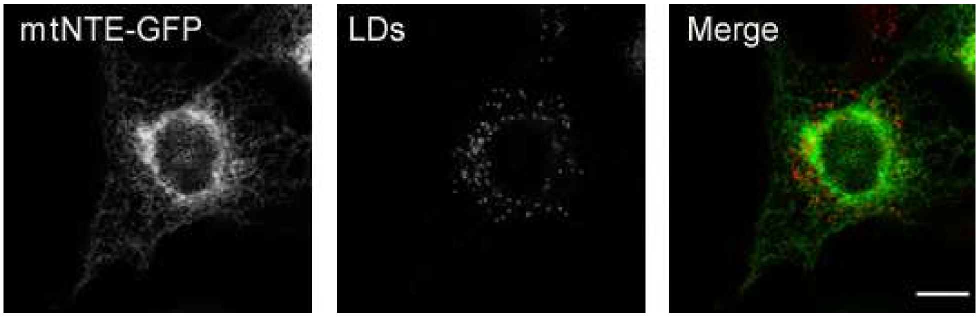

Figure 2. NTE inactive mutant mtNTE did not localize to LDs. COS-7 cells expressing mtNTE-GFP were incubated with OA overnight. LDs were labeled by LipidTOX Red. Colocalization of GFP-fused proteins with LDs was visualized by confocal laser scanning microscopy. Scale bar is10 μm. Figures are representative of three separate experiments.
